# Nodular‐type central squamous cell lung carcinoma cured by intraluminal bronchoscopic treatment: A case report

**DOI:** 10.1111/1759-7714.14090

**Published:** 2021-07-28

**Authors:** Hikaru Aoki, Keigo Uchimura, Tatsuya Imabayashi, Yuji Matsumoto, Masahiro Higashiyama, Shun‐Ichi Watanabe, Takaaki Tsuchida

**Affiliations:** ^1^ Department of Endoscopy Respiratory Endoscopy Division, National Cancer Center Hospital Tokyo Japan; ^2^ Department of Thoracic Surgery National Cancer Center Hospital Tokyo Japan; ^3^ Department of Thoracic Oncology National Cancer Center Hospital Tokyo Japan

**Keywords:** bronchial tumor, bronchoscopy, electrocautery, lung cancer, surgery

## Abstract

Primary squamous cell carcinoma (SqCC) often occurs in the trachea and bronchi. Recently, intraluminal bronchoscopic treatment (IBT) has emerged as an option for curative treatment, not just surgery, in patients with central early‐stage SqCC (CES‐SqCC). However, patients that can be cured by IBT are limited. We report a rare case of CES‐SqCC that was surgically confirmed to be cured by IBT alone. A 72‐year‐old woman had a nodular bronchial tumor at the bifurcation of right upper and intermediate bronchi. For histological diagnosis, the tumor was resected and incinerated using high‐frequency snare (HFS). Obtained specimens were diagnosed as SqCC; wedge resection of the bifurcation was performed to remove the residual lesion. However, no malignant findings were found in the excised specimens. Some patients with CES‐SqCC may be cured by aggressive diagnostic bronchoscopy. The risk of postoperative complications cannot be ignored because the surgery requires bronchoplasty in patients with CES‐SqCC. For patients with CES‐SqCC, surgical resection may be avoided by detailed assessment of residual lesion with radial probe endobronchial ultrasonography (RP‐EBUS).

## Graphical abstract

We report a rare case of central early‐stage squamous cell carcinoma (CES‐SqCC) that was surgically confirmed to be cured by intraluminal bronchoscopic treatment (IBT) alone. The risk of postoperative complications cannot be ignored because the surgery requires bronchoplasty in patients with CES‐SqCC. For patients with CES‐SqCC, surgical resection may be avoided by detailed assessment of residual lesion with radial probe endobronchial ultrasonography (RP‐EBUS).

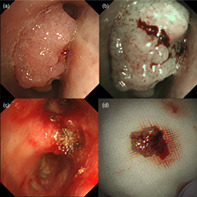

## INTRODUCTION

Primary lung squamous cell carcinoma (SqCC) often occurs in the trachea and bronchi.^1^ Smoking is a risk factor for the development of SqCC. Patients with SqCC have synchronous or multiple cancers, and many cases are difficult to operate on because of low pulmonary function caused by heavy smoking.^2^, ^3^ Intraluminal bronchoscopic treatment (IBT) such as electrocautery, argon plasma coagulation, Nd‐YAG laser, photodynamic therapy, and cryotherapy have mainly been performed as palliative treatment for obstructive airway disease.^4^ Recently, IBT has emerged as an option for curative treatment, not just surgical resection, in patients with central early‐stage SqCC (CES‐SqCC).^4^, ^5^, ^6^, ^7^

Here, we report a rare case of nodular‐type CES‐SqCC, which was resected with a high‐frequency snare (HFS) for diagnosis and was thought to have been cured by IBT alone because no malignant findings were found in the surgically obtained specimens.

## CASE REPORT

**FIGURE 1:**
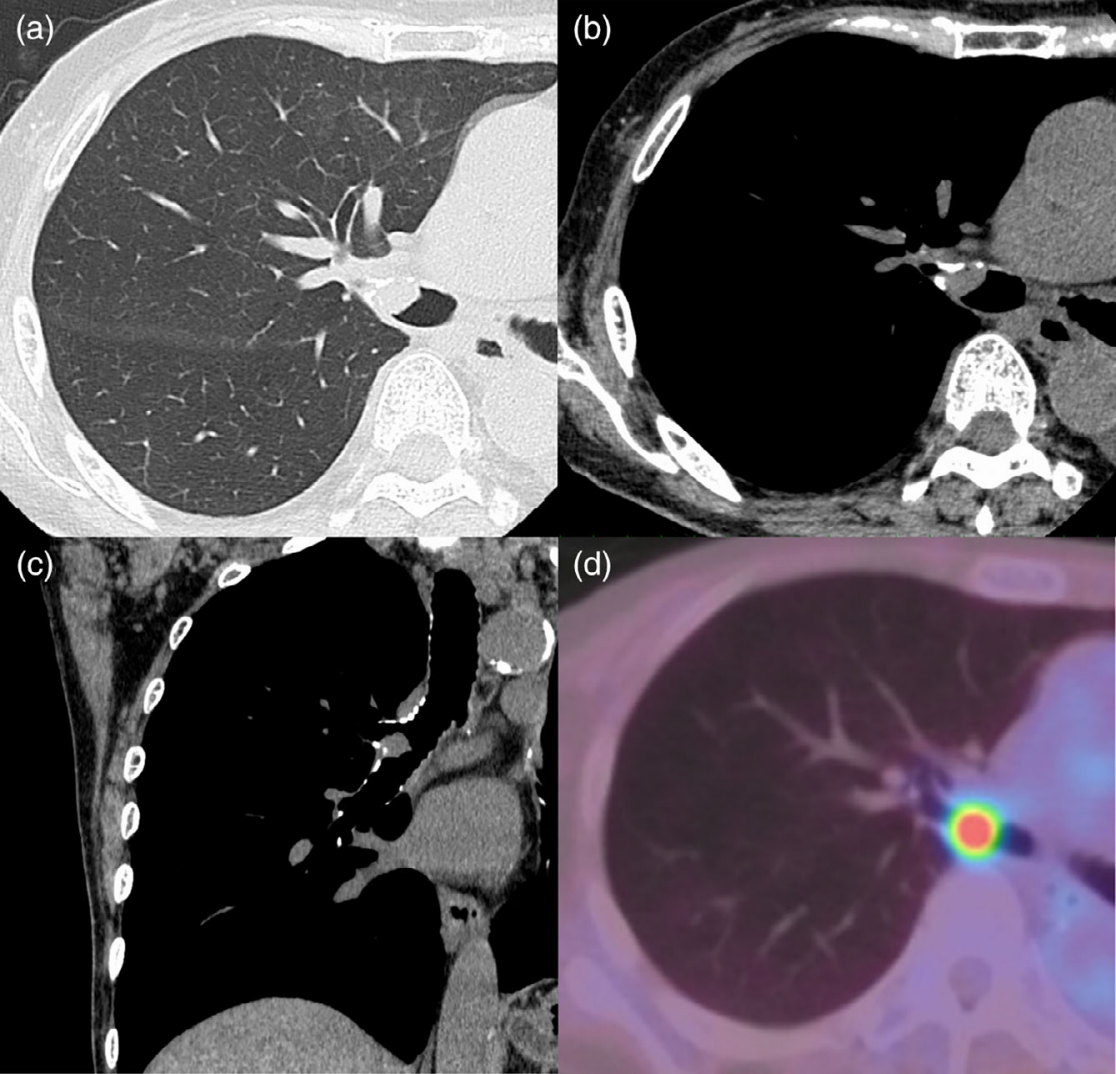
Chest computed tomography (CT) and positron emission tomography‐CT (PET‐CT). Chest CT showed a bronchial tumor with a maximum diameter of 12 mm at the bifurcation of right upper and intermediate bronchi ((a) and (b) axial image, (c) coronal image). The tumor shows high fluorodeoxyglucose (FDG) uptake with maximum standardized uptake value (SUVmax) of 7.2 on PET‐CT (d)

**FIGURE 2:**
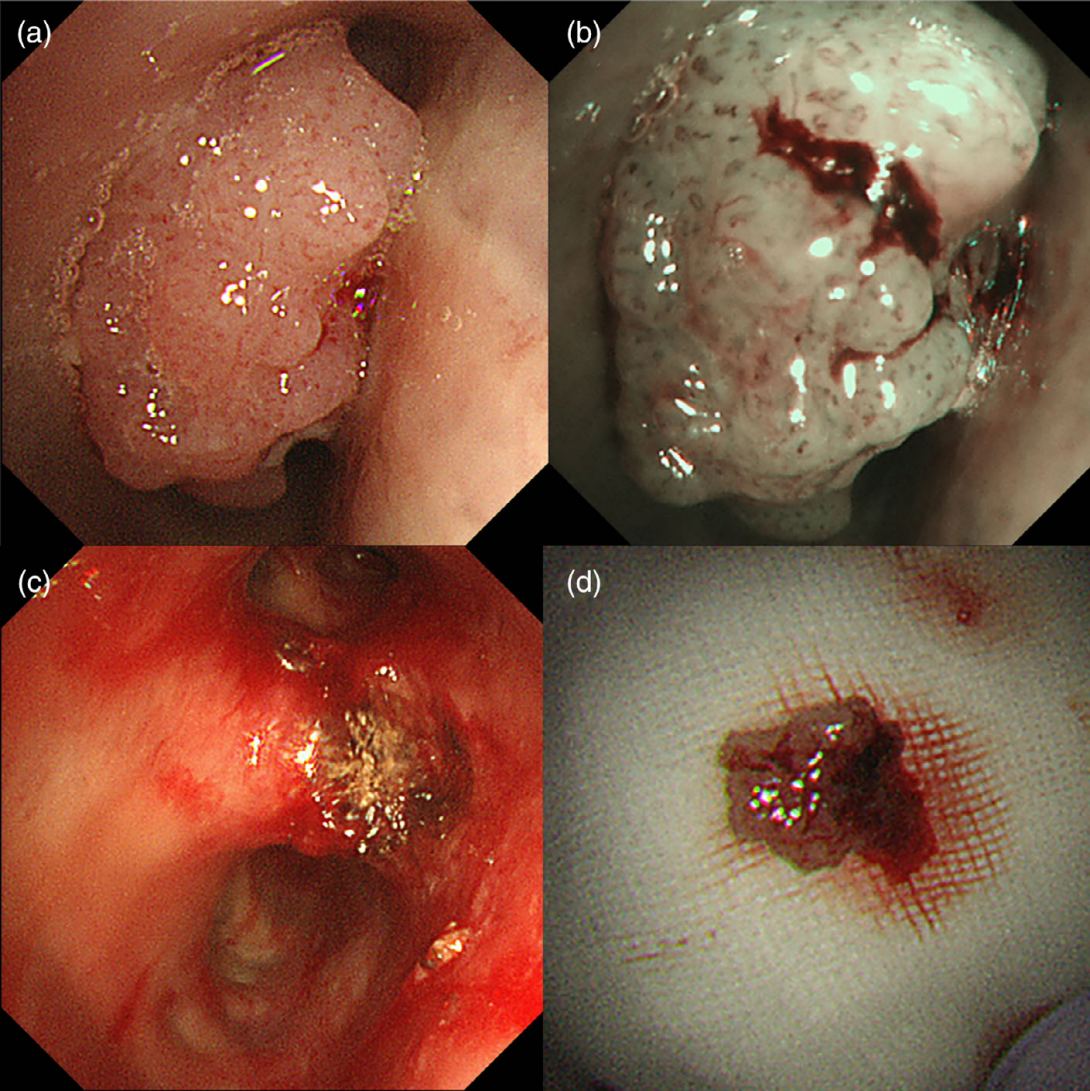
Bronchoscopic findings during white‐light and narrow band imaging (NBI) bronchoscopy. White‐light bronchoscopy shows a nodular tumor at the bifurcation of right upper and intermediate bronchi; the tumor stalk and extent could not be assessed (a). NBI bronchoscopy shows dotted blood vessels in the tumor (b). The tumor is bronchoscopically resected and the remaining tumor stalk is incinerated using a high‐frequency snare (HFS) (c). Bronchoscopic obtained specimen with HFS (d)

**FIGURE 3:**
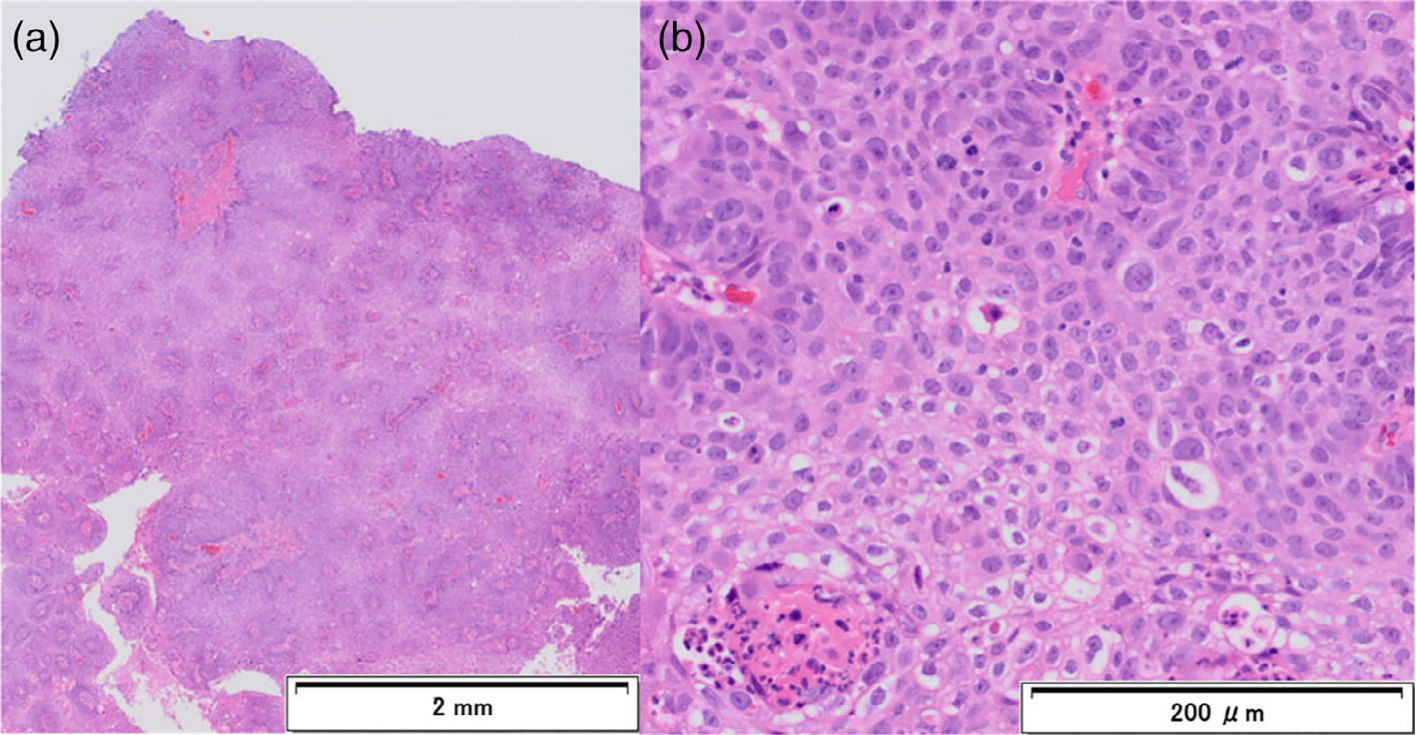
Histopathological findings of the specimens obtained by bronchoscopy. Polygonal tumor cells with partial keratinization are found, and the tumor is diagnosed as squamous cell carcinoma ((a) and (b), hematoxylin and eosin staining)

**FIGURE 4:**
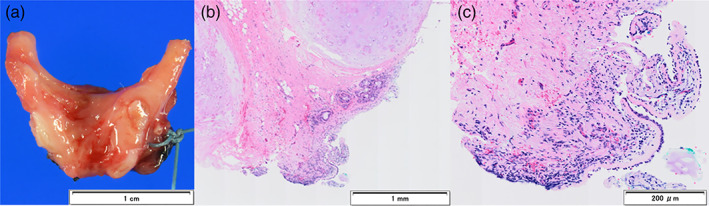
Surgically resected bronchial specimen and the histopathological findings. Resected bronchial specimens are microscopically absent of tumor ((a) the upper is bronchial wall side). Bronchial tissue shows detachment of bronchial mucosa, but no atypical epithelium or malignant cells, and some inflammatory cell infiltration and fibrosis ((b) and (c) hematoxylin and eosin staining)

A 72‐year‐old woman visited our hospital with a chief complaint of blood in sputum. She had undergone left upper sleeve lobectomy for SqCC 2 years ago. She had a smoking history of 78 pack‐years. Chest computed tomography (CT) scan showed a bronchial tumor with a maximum diameter of 12 mm at the bifurcation of right upper and intermediate bronchi (Figure 1(a)–(c)). The lesion showed high fluorodeoxyglucose (FDG) uptake with maximum standardized uptake value (SUVmax) of 7.2 on positron emission tomography (PET)‐CT (Figure 1(d)). White‐light bronchoscopy (BF‐1TQ290; Olympus) showed a nodular tumor at the bifurcation of right upper and intermediate bronchi (Figure 2(a)). Narrow band imaging bronchoscopy showed dotted blood vessels in the tumor (Figure 2(b)). Using HFS (SD‐5 L‐1; Olympus), the tumor was resected and the remaining stalk was incinerated (Figure 2(c),(d)). The tumor had not enlarged in the central or peripheral bronchi of the bifurcation. The obtained specimens were pathologically diagnosed as SqCC (Figure 3(a),(b)). Therefore, wedge resection of the bifurcation was performed 1 month after diagnostic bronchoscopy to remove the residual lesion. The extent and depth of invasion of the residual lesion were not reconfirmed preoperatively using bronchoscopy, including radial probe endobronchial ultrasonography (RP‐EBUS). There were no malignant findings in the removed bronchi and dissected right hilar lymph nodes, and only inflammatory cell infiltration and fibrosis in the bronchial tissue were found (Figure 4). No recurrence was reported for a year.

## DISCUSSION

We presented a case of nodular‐type CES‐SqCC that was cured by IBT at diagnosis. Generally, complications of bronchoplasty are caused by anastomotic problems.^8^ The morbidity and mortality for wedge resection of the bronchus are reported to be 11.8% and 5.9%, respectively; higher than those of IBT.^9^ Here, chest high‐resolution CT (HR‐CT) did not reveal lymph node metastasis or invasion beyond the cartilaginous layer (CL), but the size and bronchoscopic findings suggested invasion within the CL. Therefore, we performed surgery.

When considering curative treatment with IBT for CES‐SqCC, accurate evaluation is essential for size, the central or peripheral extent, the presence of lymph node metastasis, and the depth of bronchial wall invasion.^5^ A meta‐analysis reported that autofluorescence and narrow band imaging (NBI) bronchoscopy has a detection sensitivity of 88%–100% for early cancer lesions, compared to 50%–62% for white‐light bronchoscopy, and are widely used to detect the tumor extent.^10^ CES‐SqCC can be divided into three types (hypertrophic‐type, nodular‐type, and polypoid‐type) based on bronchoscopic findings. The larger lesions are known to have deeper invasion within bronchial wall.^11^, ^12^ A retrospective study using surgical specimens of CES‐SqCC showed that 82.9% (29/35) of the lesions with a diameter larger than 10 mm or more invaded the CL; this is difficult to cure bronchoscopically. They also reported that 94.1% (16/17) of the nodular‐type CES‐SqCC invaded the CL.^11^

Recent guidelines recommend curative IBT only for patients for whom surgery is not indicated^13^; the effectiveness of IBT for undetectable CES‐SqCC on HR‐CT has been widely reported.^14^ Surgery is superior to bronchoscopy because the entire tumor and lymph node specimens can be provided for diagnosis. However, low pulmonary function because of heavy smoking and simultaneous or multiple occurrences makes surgical resection difficult in patients with CES‐SqCC.^2^, ^3^ The depth of tissue necrosis caused by electrocautery involving HFS in airway lesions has been reported to be several millimeters.^7^, ^15^, ^16^ Therefore, IBT is reported to be curatively inappropriate if HR‐CT shows thickening of the bronchial wall or extension of the tumor into the peribronchial region.^17^, ^18^

Although not used in this case, several studies have attempted to use RP‐EBUS to determine depth of invasion of CES‐SqCC before IBT.^19^, ^20^ One prospective study used RP‐EBUS to assess whether the depth of tumor invasion reached the CL; an accuracy rate of 80.0% was reported.^20^ Therefore, regardless of bronchoscopic features or size of the lesion, bronchoscopists should consider the need for surgery. Evaluation of the depth of tumor invasion using RP‐EBUS before and after IBT would have been helpful in deciding whether to perform additional treatment.

In conclusion, we report a case of surgically confirmed CES‐SqCC that was completely cured by IBT alone. When considering surgery for residual lesions after IBT, non‐surgical options should be considered by assessing not only the extent but also the depth of invasion using bronchoscopy, including RP‐EBUS.

## DISCLOSURE

The authors declare that they have no competing interests.
